# Study on the Process and Mechanism of Preparing Lanthanum Carbonate from Rare Earth Chloride Solution

**DOI:** 10.3390/ma19081645

**Published:** 2026-04-20

**Authors:** Binru Xu, Wenli Lu, Meng Wang, Chunguang Song, Xin Sun, Yanyan Zhao

**Affiliations:** 1 National Engineering Research Center for Rare Earth, Grirem Advanced Materials Co., Ltd., Beijing 100088, China; xubinru@grirem.com (B.X.); sunxin@grirem.com (X.S.); 2 General Research Institute for Nonferrous Metals, Beijing 100088, China; 3 GRIMAT Engineering Institute Co., Ltd., Beijing 101407, China; 4 State Key Laboratory of Critical Metals Beneficiation, Metallurgy and Purification, Beijing 100088, China; zhaoyanyan@grirem.com

**Keywords:** rare earth carbonates, dissolution-reprecipitation, CO_2_ carbonation, green

## Abstract

To address the issues of high wastewater treatment costs and the lack of recycling associated with conventional precipitants such as oxalic acid and ammonium bicarbonate in rare earth precipitation processes, this study proposes a novel gradient alkali conversion–carbonation method based on a green process coupling “rare earth chloride alkali conversion-carbonation with sodium chloride electrolysis.” The primary scientific objective is to elucidate the crystallization mechanism and to achieve controlled preparation of high-quality lanthanum carbonate. By gradient-controlling the addition sequence and rate of alkali liquor and CO_2_, lanthanum carbonate tetrahydrate was successfully synthesized. Characterization by XRD, SEM, ICP, and laser particle size analysis indicates that the product prepared by the gradient alkali conversion–carbonation method exhibits a single phase with high crystallinity, as evidenced by sharp and clear XRD diffraction peaks. Furthermore, the median particle size of the product obtained via this method is relatively large, reaching approximately 10 μm, while the particle size distribution Span value remains around 1.0. Mechanistic studies suggest that this method effectively regulates the crystallization process by precisely controlling the introduction and slow dissolution of the La(OH)_3_ precursor, thereby reducing the supersaturation of the system during carbonation and facilitating the dissolution–reprecipitation of La^3+^. This work provides a theoretical basis for the preparation of high-quality rare earth carbonates and a process reference for the green recycling route.

## 1. Introduction

Rare earth elements (REEs) possess unique 4f electronic structures that endow them with excellent optical, electrical, magnetic, and catalytic properties, making them critical materials for strategic industries such as high-end manufacturing and new energy [1,2,3,4,5,6]. China has established a world-leading system for rare earth extraction and separation [7,8]. However, the precipitation step in rare earth oxide production commonly uses precipitants such as oxalate, ammonium bicarbonate, and sodium carbonate, which generate wastewater that is difficult to recycle, causing severe environmental concerns [9,10,11,12]. Although magnesium bicarbonate [13,14] has been explored to enable magnesium recycling, it still produces calcium chloride as a byproduct and does not achieve a closed-loop material flow across the entire process. Therefore, developing a green, low-carbon, and resource-recyclable precipitation process for rare earths is urgently needed for sustainable industrial development. However, beyond the environmental challenge, a more fundamental scientific issue has been largely overlooked: the crystallization mechanism of rare earth carbonates under CO_2_ carbonation conditions remains poorly understood. In particular, how supersaturation evolves and how nucleation/growth are kinetically controlled are key knowledge gaps that limit process optimization.

The “alkali conversion–carbonation of rare earth chloride coupled with NaCl electrolysis” process proposed by Professor Xiaowei Huang’s team offers a complete closed-loop solution: NaOH precipitates the rare earth while CO_2_ is introduced for carbonation, yielding rare earth carbonate and an NaCl filtrate; the filtrate is electrolyzed to regenerate NaOH for reuse, while the byproducts Cl_2_ and H_2_ are combined to produce HCl for upstream operations, thereby achieving full Na/Cl recycling. The core of this process is CO_2_ carbonation precipitation. However, existing carbonation studies generally adopt the “synchronous alkali conversion–carbonation” method (i.e., simultaneous addition of NaOH and CO_2_) [15,16,17,18] and suffer from two critical issues:

(1) Insufficient attention to precipitation yield. Most studies focus on morphological control of the product, while the precipitation yield—a key parameter for industrial production—has been largely overlooked. For instance, the rare earth precipitation yield in [15] is only 88.83%, and yields are not reported in [16,17,18]. Moreover, no study to date has described complete rare earth precipitation.
(2) Unclear carbonation crystallization mechanism. Although some researchers have speculated that CO_2_ carbonation might improve crystal growth via a “quasi-homogeneous precipitation” mechanism, this concept remains at the hypothetical stage in rare earth solution carbonation systems, lacking direct experimental evidence such as intermediate species evolution or real-time supersaturation measurements. The synchronous addition of NaOH and CO_2_ tends to generate an instantaneous high supersaturation that triggers explosive nucleation; however, the specific regulatory pathways and crystallization kinetics remain poorly understood.

The researchers’ study on the carbonation process of rare earth chloride solution is shown in Table 1.

To address these issues within the green closed-loop process framework, this work proposes a novel gradient alkali conversion–carbonation method: first, a portion of La^3+^ is converted into a La(OH)_3_ precursor; then, the remaining alkali and CO_2_ are introduced simultaneously, exploiting a “dissolution–reprecipitation” process of the precursor to reduce supersaturation during carbonation. This study aims to elucidate the microscopic mechanism by which this process controls crystallization and to achieve complete rare earth precipitation as well as controllable preparation of high-quality lanthanum carbonate at conventional industrial concentrations, thereby providing a theoretical basis for the scale-up of the green recycling process.

## 2. Materials and Methods

### 2.1. Raw Materials

All reagents used in this experiment are from China; see Table 2 for details.

**Figure 1:**
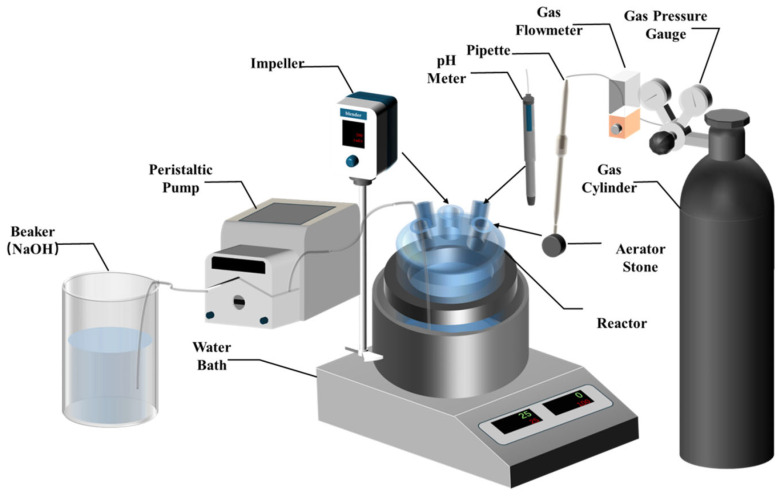
Experimental setup.

The main equipment used in the experiment is shown in Figure 1.

### 2.2. Research Methods

The following general conditions apply to both precipitation methods. Lanthanum oxide (4N, Ganzhou High-tech Industrial Development Zone, Ganzhou City, Jiangxi Province, China) was dissolved in hydrochloric acid to prepare a 1.0 mol/L lanthanum chloride feed solution with a pH of 3. A 2 mol/L sodium hydroxide solution was used as the pH regulator. The reaction temperature was maintained at 25 °C using a constant-temperature water bath, and stirring was controlled by an electric stirrer (Model “GZ120-S”, Shanghai Leigu Instrument Co., Ltd., Shanghai, China). In each experiment, 200 mL of the lanthanum chloride solution was placed in a 2.5 L reactor. After the reaction, the mixture was filtered and thoroughly washed with stirring, and the filter cake was dried at 60 °C for 48 h to obtain the lanthanum carbonate precipitate.

#### 2.2.1. Synchronous Alkali Conversion–Carbonation

**Figure 2:**
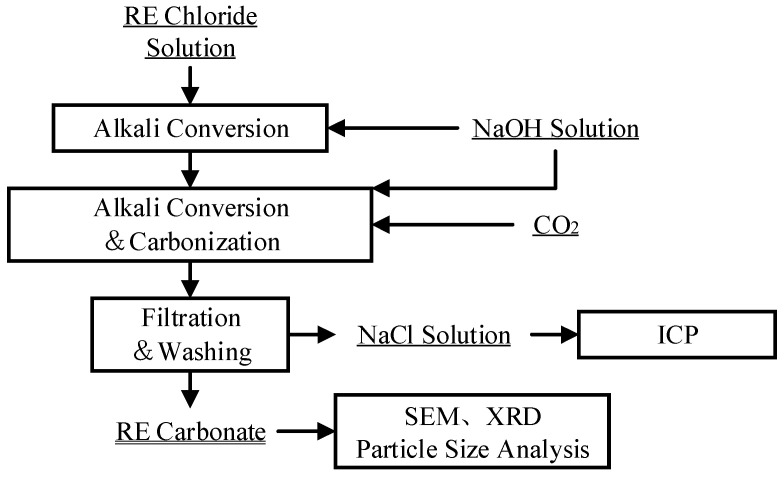
Flowchart of gradient alkali conversion and carbonation.

In this method, sodium hydroxide solution was added at a constant rate over 30 min using a peristaltic pump (Model “SENZPUMP 310D”, SENZ Co., Ltd., Baoding, Hebei, China). Simultaneously, CO_2_ was introduced into the solution at a flow rate of 2 L/min for 60 min. The process flow is similar to that shown in Figure 2, except that the NaOH addition and CO_2_ sparging are performed concurrently from the beginning.

#### 2.2.2. Gradient Alkali Conversion–Carbonation

The process consists of two stages (Figure 2). First, for alkali conversion, sodium hydroxide solution was added at a constant rate over 30 min using a peristaltic pump to form a lanthanum hydroxide precursor. Second, for carbonation, the remaining sodium hydroxide solution was added at a constant rate over 60 min while simultaneously introducing CO_2_ at a flow rate of 2 L/min.

#### 2.2.3. Material Characterization

The morphology of the powders was characterized using a field emission scanning electron microscope (JSM-7900F, JEOL Ltd., Tokyo, Japan). Particle size analysis was conducted via laser diffraction particle size analysis (LA-960, HORIBA, Ltd., Kyoto, Japan). Phase composition was determined using an X-ray diffractometer (Smartlab, Rigaku Co., Ltd., Tokyo, Japan) with continuous scanning over a range of 5° to 90°. The chemical composition of the solutions and precipitates was analyzed by inductively coupled plasma optical emission spectrometry (ICP-OES, Optima 8300, PerkinElmer, Waltham, MA, USA).

#### 2.2.4. Thermodynamic Simulation

**Figure 3:**
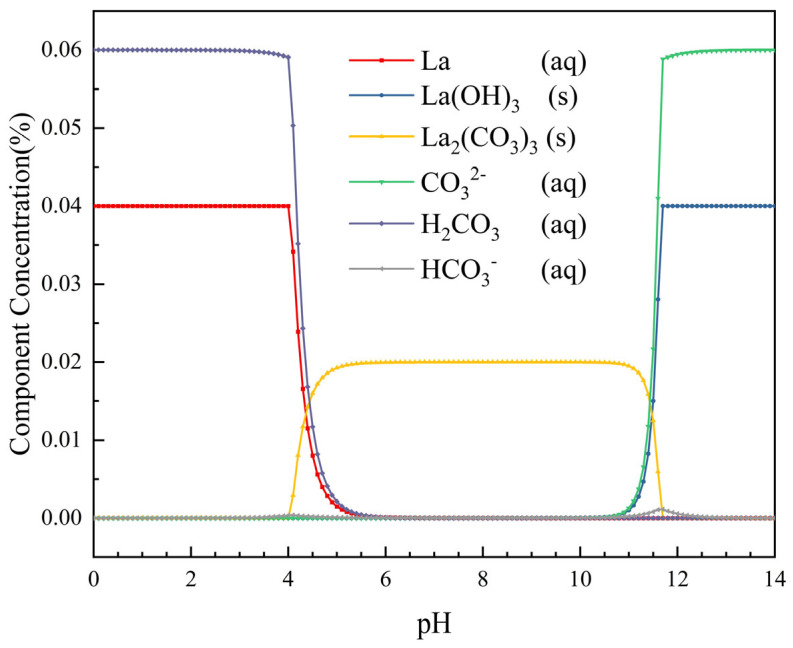
Species mole fractions as a function of pH in the carbonation system of lanthanum chloride solution (simulated by Visual MINTEQ at 25 °C, [La^3+^] = 0.04 Molal, DIC (Dissolved Inorganic Carbon) = 0.06 Molal).

To guide the pH control strategy, the speciation of the LaCl_3_-CO_2_-H_2_O system was simulated using Visual MINTEQ 4.0. However, direct simulation of the experimental 1.0 mol/L LaCl_3_ solution is not feasible because the high ionic strength exceeds the reliable range of the NIST thermodynamic database. Therefore, a diluted concentration (0.04 molal LaCl_3_) was used to obtain a qualitative trend of species transformation. The simulation conditions were 25 °C, CO_2_ partial pressure 0.1 atm, DIC = 0.06 molal, pH 2–14. The results (Figure 3) show that La_2_(CO_3_)_3_ is stable at pH 6–10, while La(OH)_3_ forms above pH 10. This supports the two-step design: first form La(OH)_3_ at high pH, then convert it to La_2_(CO_3_)_3_ by lowering pH. The quantitative pH boundaries should be considered approximate; the actual pH window was verified by experimental phase analysis (Section 3.2 and Section 3.3).

## 3. Results

### 3.1. Speciation Analysis of the Carbonation System in Chloride Rare Earth Feed Solution

Figure 3 presents the species distribution of LaCl_3_ solution during the carbonation process, as simulated using the Visual MINTEQ software. As shown in Figure 3, within the carbonation system, lanthanum carbonate is preferentially generated when the solution pH rises above 4 and remains stable within the pH range of 6–10. When the pH exceeds 10, lanthanum hydroxide precipitation begins to appear, and the content of lanthanum carbonate gradually decreases. It can be inferred from this that lanthanum hydroxide can be preferentially generated under high-pH conditions and subsequently converted to lanthanum carbonate by lowering the pH of the solution. Theoretically, during this process, NaOH and CO_2_ can be added in a gradient manner to control the solution pH, thereby regulating the reaction rate of the conversion from lanthanum hydroxide to lanthanum carbonate. This allows for the slow release of La^3+^, achieving the purpose of dissolution–reprecipitation, reducing the supersaturation of the system, and achieving an effect similar to homogeneous precipitation [19,20,21].

To avoid potential artifacts from high ionic strength in the thermodynamic database, a diluted concentration (0.04 molal) was used for speciation simulation; this provides a qualitative pH-dependent trend that guides the experimental design.

### 3.2. Preparation of Lanthanum Carbonate by Synchronous Alkali Conversion Carbonation

To investigate the practical effect of simultaneous alkali conversion and carbonation, both the precipitated product obtained through carbonation and the resulting solution were analyzed. In this process, experimental groups with sodium hydroxide addition volumes of 300, 320, 340, and 360 mL—based on the theoretical alkali dosage—were designated as Syn-1, Syn-2, Syn-3, and Syn-4, respectively.

**Figure 4:**
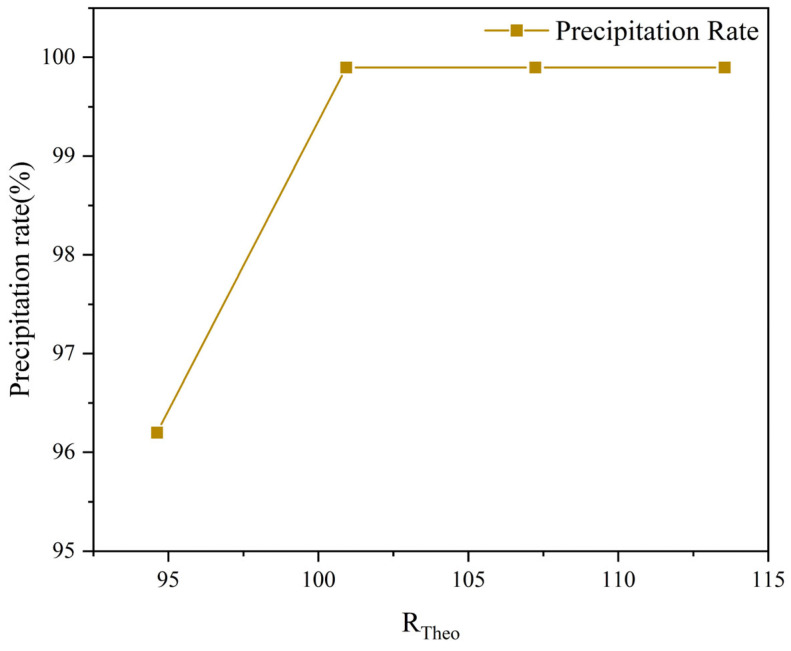
Relationship between the R_Theo_ (ratio of actual to theoretical NaOH addition) and precipitation rate.

According to the theoretical calculations mentioned above, the volume of sodium hydroxide solution added serves as a key parameter for regulating the pH of the solution and must be strictly controlled to ensure complete precipitation of the rare earth feed solution. Figure 4 illustrates the relationship between the volume of sodium hydroxide added throughout the entire process and the precipitation rate of La^3+^. As shown in the figure, the required volume of NaOH solution for complete precipitation of La^3+^ is 317 mL.

According to the reaction

$$La^{3+}+3OH^{-}=La{\left(OH\right)}_{3}$$

Actual LaCl_3_ concentration = 1.056 mol/L, volume = 200 mL → n(La^3+^) = 0.2112 mol. Stoichiometric ratio (La^3+^:OH^−^ = 1:3) requires 316.8 mL of 2 mol/L NaOH (rounded to 317 mL). Filtrate volume was recorded after filtration, residual La^3+^ concentration was measured by ICP-OES, and the precipitation rate was calculated by mass balance. Syn-1 (300 mL NaOH): residual La^3+^ = 1.9 g/L, filtrate volume = 661 mL, which indicates precipitation rate = 96.20%; Syn-2–4 (≥320 mL NaOH): residual La^3+^ = ppm level (<10 mg/L), which indicates precipitation rate ≥ 99.99%.

When the volume of sodium hydroxide solution added was below the theoretical value, the precipitation rate was only 96.20%, and a certain amount of La^3+^ remained in the filtrate. This result indicates, on one hand, that the rare earths are difficult to recover completely; on the other hand, it leads to a high residual La^3+^ content in the filtrate, making it challenging to recycle the filtrate for the membrane electrolysis process. When the added volume was at or above the theoretical value, the precipitation rate reached ≥99.99%, with La^3+^ being almost completely precipitated in the form of lanthanum carbonate.

**Figure 5:**
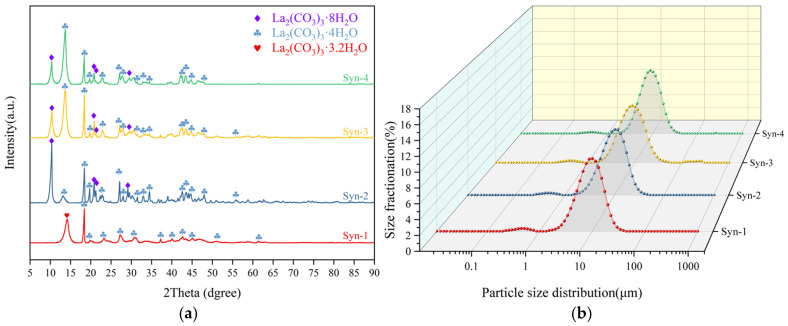
Analysis of products from synchronous alkali conversion and carbonation: (**a**) XRD pattern; (**b**) Laser particle size distribution.

Phase analysis of the products from the synchronous alkali conversion–carbonation process, as shown in Figure 5a, revealed that at a low NaOH addition level (Syn-1), the product phases primarily consisted of a mixture of lanthanum carbonate tetrahydrate (La_2_(CO_3_)_3_·4H_2_O, PDF#006-0076) and lanthanum carbonate octahydrate (La_2_(CO_3_)_3_·8H_2_O, PDF#025-1400), with a small amount of diffraction peaks corresponding to La_2_(CO_3_)_3_·3.2H_2_O also appearing. This particular phase was first reported in the study by Xiao et al. [22]. When an excessive amount of alkali was added, the product was mainly a mixture of lanthanum carbonate tetrahydrate (La_2_(CO_3_)_3_·4H_2_O, PDF#006-0076) and lanthanum carbonate octahydrate (La_2_(CO_3_)_3_·8H_2_O, PDF#025-1400). The above results indicate that the phase distribution of the products from the synchronous alkali conversion–carbonation process is relatively complex, which is detrimental to the subsequent preparation of high-quality rare earth oxides.

Furthermore, the particle size distribution of the precipitated products from the synchronous alkali conversion process was analyzed. As shown in Figure 5b, the median particle sizes (D50) for Syn-1, Syn-2, Syn-3, and Syn-4 were 8.42, 8.79, 8.03, and 8.10 μm, respectively. The corresponding particle size distribution span (Span) values were 1.19, 1.20, 1.32, and 1.22, respectively, with all Span values exceeding 1.19. Each experimental group exhibited a bimodal peak in the particle size distribution, attributed to a fine particle fraction, indicating non-uniform particle sizes. The formula for calculating the Span value is

(1) $$Span=\frac{D_{90}-D_{10}}{2D_{50}}$$

**Figure 6:**
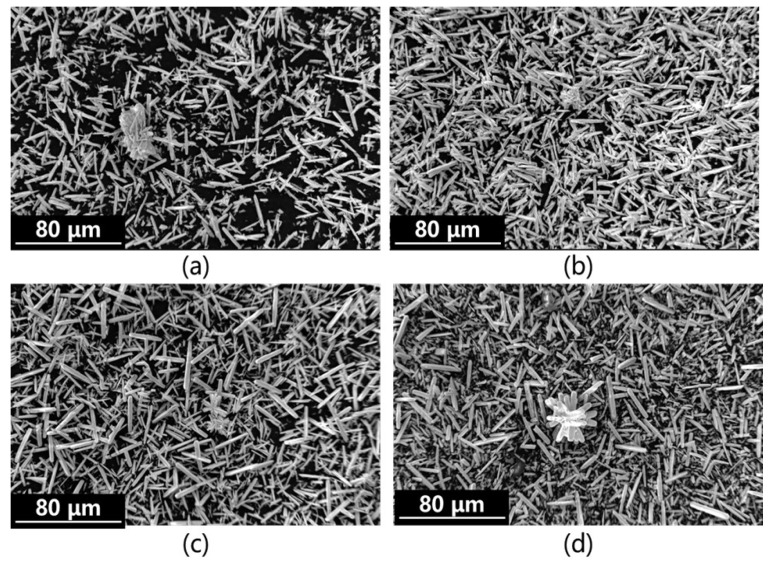
Morphology analysis of precipitates from simultaneous alkali conversion and carbonation: (**a**) Syn-1; (**b**) Syn-2; (**c**) Syn-3; (**d**) Syn-4.

As shown in Figure 6, the micromorphology of the product obtained via simultaneous alkaline conversion further confirms its non-uniform particle size distribution, presenting as elongated needle-like or rod-shaped crystals. A small number of these crystals form spiky spherical secondary particles, which further substantiates the existence of a small particle size peak in the simultaneous alkaline conversion and carbonation process.

**Figure 7:**
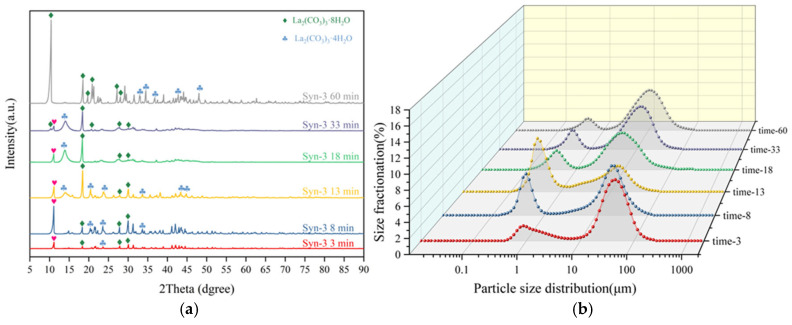
Time-dependent analysis of the synchronous alkali conversion and carbonation process: (**a**) Evolution of XRD patterns; (**b**) Corresponding evolution of particle size distribution. The red heart symbol indicates that the phase of lanthanum carbonate tetrahydrate has shifted within 1 degree.

To further investigate the phase transition mechanism during the simultaneous alkali conversion–carbonation process, time-sampling analysis was conducted on the Syn-3 group. Figure 7a presents the XRD patterns of the products obtained at different time intervals during the simultaneous alkali conversion–carbonation process. The results indicate that a mixed phase of La_2_(CO_3_)_3_·4H_2_O and La_2_(CO_3_)_3_·8H_2_O appears at the initial stage of the reaction (3 min). As the reaction proceeds, only the diffraction peak intensities of the phases change, while the phase composition remains essentially unchanged, suggesting that the core process in the subsequent stages is the nucleation and growth of existing grains. During the first 30 min of the simultaneous alkali conversion process, the low pH of the system results in low supersaturation, leading to slow nucleation and sufficient crystal growth; thus, the thermodynamically stable phase La_2_(CO_3_)_3_·4H_2_O is the only phase present [23]. However, when the amount of sodium hydroxide added exceeds the theoretical value, diffraction peaks corresponding to La_2_(CO_3_)_3_·8H_2_O appear in the product phase of the simultaneous alkali conversion process. This is attributed to the excessive sodium hydroxide causing a high pH in the solution, which increases the supersaturation of the system and disrupts the rate balance between nucleation and crystal growth [24], simultaneously activating the formation window for the metastable phase La_2_(CO_3_)_3_·8H_2_O. Since the environmental conditions during the subsequent grain growth stage do not change in a way that favors phase transition, this mixed phase is stably retained until the end of the reaction. Figure 7b displays the particle size distribution results of the products obtained from time-sampling during the simultaneous alkali conversion–carbonation and gradient alkali conversion–carbonation processes. Throughout the entire simultaneous alkali conversion–carbonation process, a bimodal distribution representing small crystal nuclei and large particles persists, which is attributed to the uncontrollable rates of nucleation and crystal growth, resulting in a broad particle size distribution of the final product.

**Figure 8:**
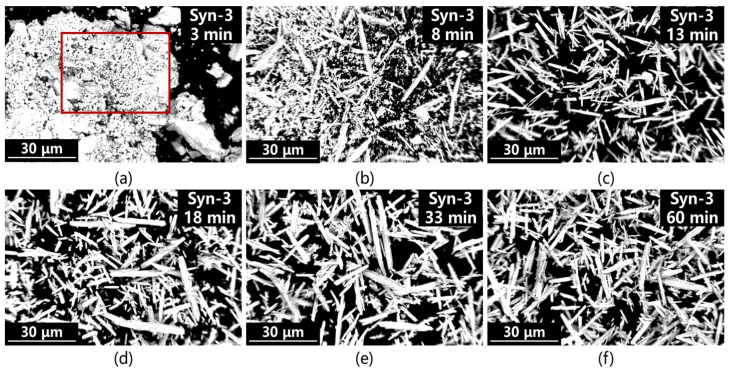
SEM analysis images of the Syn-3 group in the simultaneous alkali conversion and carbonation process at different time points: (**a**) 3 min; (**b**) 8 min; (**c**) 13 min; (**d**) 18 min; (**e**) 33 min; (**f**) 60 min. The red square indicates the rapidly formed primary crystal nuclei.

Figure 8 presents the SEM images of products sampled at different time points during the simultaneous alkali-to-carbonate conversion process. As shown in Figure 8a, a large number of crystal nuclei are formed rapidly in the initial stage; Figure 8b shows that nucleation and crystal growth occur simultaneously; Figure 8c,d mainly exhibit crystal growth, a large number of fine microcrystals emerge at the initial stage of the reaction, indicating an almost explosive nucleation process. Subsequently, these nuclei rapidly develop into needle-like whiskers. It is noteworthy that nucleation and growth occur concurrently throughout the entire reaction process, and neither rate can be precisely controlled. This kinetic uncontrollability directly leads to a complex phase composition and a wide particle size distribution in the final product, as well as the appearance of peak splitting in the corresponding particle size patterns.

**Figure 9:**
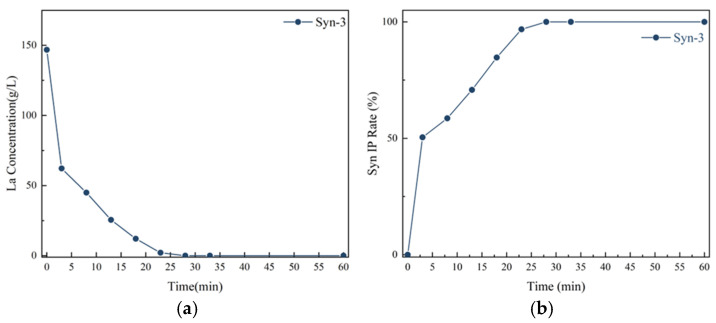
Time-dependent analysis of the synchronous alkali conversion and carbonation process: (**a**) Real-time concentration of lanthanum ions; (**b**) Real-time precipitation rate.

Furthermore, the filtrate components were analyzed using ICP, and the results are shown in Figure 9. At the initial stage of the reaction, the La^3+^ concentration was extremely high but decreased sharply as the reaction proceeded. Figure 9b illustrates the variation in the instantaneous precipitate rate, which increased sharply within a short period. This trend is closely linked to the substantial consumption of solutes during the rapid nucleation stage. This phenomenon reveals the underlying reason for phase mixing and the broad particle size distribution, i.e., that the excessively high supersaturation leads to a complex nucleation process.

It should be noted that Figure 9 presents only the results for group Syn-3. This is because the NaOH addition in Syn-3 is exactly the theoretical value (317 mL) under which La^3+^ precipitation is most complete and the reaction process is most representative. The real-time concentration and precipitation rate trends for the under-base groups (Syn-1, Syn-2) and the over-base group (Syn-4) are qualitatively consistent with those of Syn-3; their endpoint data are already summarized in Figure 5 and are therefore not repeated here.

The calculation formula for the real-time precipitation(R_IP_) rate is as follows:

(2) $$R_{IP}=\left(1-\frac{c_{x}\rho_{x}V_{x}}{M_{x}}\right)\;*\;100\%$$

(3) $$M_{x}=M_{x-1}-c_{x-1}\rho_{x-1}v$$

In the calculation formula for the real-time precipitation rate, c_x_ is the mass fraction of La element in the filtrate at the x-th sampling; ρₓ is the density of the filtrate at the x-th sampling, approximately 1 × 10^3^ kg/m^3^; V_x_ is the volume of the slurry at the x-th sampling, roughly equal to the volume of the solution in the system; M_x_ is the total mass of La element in the system at the x-th sampling; and v is the volume of slurryremoved.

Based on the phase, morphological characteristics, and particle size distribution of the intermediate products, it is evident that the rapid nucleation during the simultaneous alkali conversion and carbonation process is the primary cause of the non-uniform phase, broad particle size distribution, and acicular morphology of the product. The excessively high supersaturation of the system triggers instantaneous and concentrated nucleation at the initial reaction stage, disrupting the equilibrium of the reaction system and resulting in poor-quality precipitates. Therefore, controlling this nucleation process is crucial for optimizing product performance. Inspired by these findings, this study proposes a novel stepwise carbonation method, as described below.

### 3.3. Preparation of Lanthanum Carbonate via Gradient Alkali Conversion–Carbonation

#### 3.3.1. Discussion on the Gradient Alkali Conversion–Carbonation Process

Consistent with the simultaneous alkaline conversion carbonation, the experimental groups with gradient alkaline conversion carbonation using sodium hydroxide volumes of 300, 320, 340, and 360 mL were designated as Gra-1, Gra-2, Gra-3, and Gra-4, respectively.

Gra-1 (300 mL NaOH): residual La^3+^ = 1.9 g/L, filtrate volume = 659 mL, which indicates precipitation rate = 96.18%; Gra-2–4 (≥320 mL NaOH): residual La^3+^ = ppm level, which indicates precipitation rate ≥ 99.99%.

**Figure 10:**
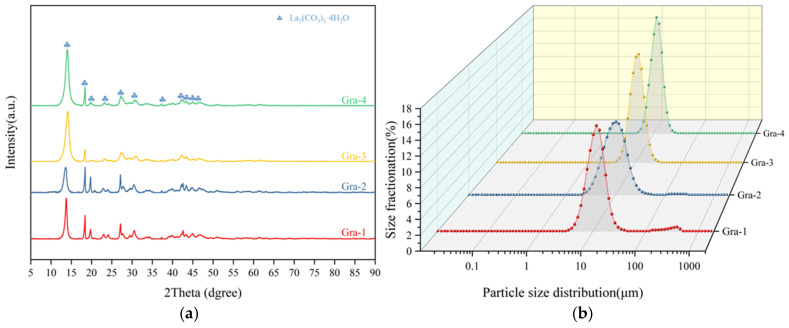
Analysis of products from gradient alkali conversion and carbonation: (**a**) XRD pattern; (**b**) Laser particle size distribution.

Similarly, phase analysis of the precipitation products from gradient alkaline conversion carbonation, as shown in Figure 10a, revealed that the amount of sodium hydroxide added had little effect on the phase composition of the final products, all of which were a single phase of La_2_(CO_3_)_3_·4H_2_O. Compared to the products of simultaneous alkaline conversion carbonation, gradient alkaline conversion carbonation yielded precipitation products with higher phase purity, attributed to the controlled release of La^3+^ during the carbonation process, which resulted in an effect similar to homogeneous precipitation [19,20,21].

As shown in Figure 10b, the particle size distribution curve obtained by the gradient alkali conversion and carbonation process exhibits a sharp, symmetric single peak, indicating that the product consists of uniformly sized, well-dispersed particles.

The D50 values for the Gra-1, Gra-2, Gra-3, and Gra-4 groups of gradient alkaline conversion carbonation were 10.78, 9.05, 10.00, and 10.84 μm, respectively, with Span values of 1.15, 1.22, 1.08, and 1.06. The particle size distribution curves showed no obvious peak splitting. Comparison reveals that the particle size distribution curves of the products obtained by gradient alkaline conversion were narrower, with no obvious secondary peaks, indicating good particle size distribution performance. Among them, the Gra-3 and Gra-4 groups showed the most significant improvement. The distribution curves of the products from gradient alkaline conversion exhibited sharp, symmetrical single peaks, indicating that the products consisted of uniformly sized and well-dispersed particles. This is attributed to the dissolution–reprecipitation effect during the gradient alkaline conversion process, which enabled the controlled release of La^3+^, ensuring a low supersaturation level in the system and effectively controlling the nucleation and crystal growth of the products.

**Figure 11:**
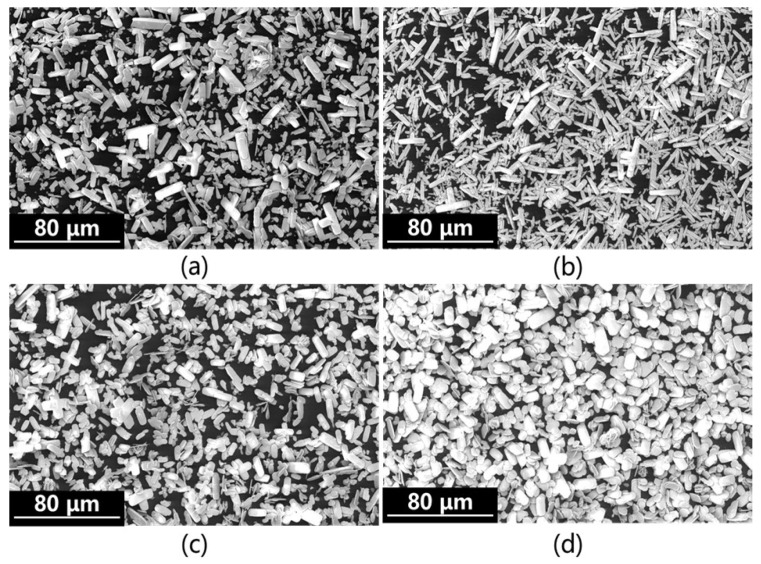
Morphology analysis of precipitates from gradient alkali conversion and carbonation: (**a**) Gra-1; (**b**) Gra-2; (**c**) Gra-3; (**d**) Gra-4.

The microstructure of the gradient alkali-converted carbonation product was observed and analyzed, with the results shown in Figure 11, which further corroborates the existence of the La^3+^ dissolution–reprecipitation process and indicates that the particle size distribution of the product tends to be uniform. As can be seen from Figure 11a–d, the obtained crystals exhibit a spindle-like flaky morphology with smooth surfaces and clear boundaries; the particles are independent of each other, with no obvious agglomeration observed.

#### 3.3.2. Analysis of the Gradient Alkali Conversion–Carbonation Mechanism

**Figure 12:**
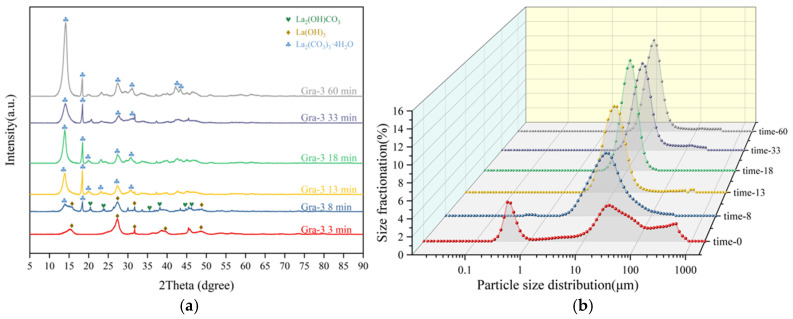
Time-dependent analysis of the gradient alkali conversion and carbonation process: (**a**) Evolution of XRD patterns; (**b**) Corresponding evolution of particle size distribution.

To gain deeper insight into the phase transition mechanism during the gradient alkaline conversion–carbonation process, time-dependent sampling analysis was conducted on the Gra-3 group. It should be noted that the 0 min sample in this time series (i.e., Figure 12a and the initial point in Figure 12b) represents the final morphology of the lanthanum hydroxide precursor obtained after the alkali conversion step and just before carbonation. This study presents these data together with the subsequent carbonation data in a comparative manner to show the complete evolution from precursor to product; therefore, no separate section is devoted to the precursor alone.

Figure 12a presents the XRD results of the products obtained at different time points throughout the process. The results indicate that at the initial stage of the carbonation reaction (0 min), the phase is exclusively La(OH)_3_ (PDF#06-0585). After 8 min of reaction, diffraction peaks corresponding to La(OH)CO_3_ (PDF#49-0981) appear, along with the incipient formation of some La_2_(CO_3_)_3_·4H_2_O (PDF#06-0076). By 13 min, the precursor phase has completely transformed into La_2_(CO_3_)_3_·4H_2_O; thereafter, only the peak intensity increases with enhanced crystallinity. Tracking the reaction process of Gra-3 demonstrates that the conversion does not proceed in a single step but follows the pathway La(OH)_3_ → La(OH)CO_3_ (intermediate phase) → La_2_(CO_3_)_3_·4H_2_O. It should be acknowledged that this pathway and the subsequent interpretation regarding supersaturation control are currently inferred from the XRD, SEM, and ICP results in combination with literature analogies [23,24]; direct confirmation by in situ pH monitoring, direct supersaturation measurement, or higher-resolution in situ characterization is needed in future work.

Meanwhile, as shown in Figure 12b, analysis of the particle size distribution during the gradient alkaline conversion–carbonation process reveals that in the initial stage (0 min), lanthanum hydroxide agglomerates exhibit a multimodal distribution. However, as the reaction proceeds, the lanthanum hydroxide gradually dissolves, and the particle size distribution rapidly converges to a single sharp peak centered at ~10 μm, indicating a highly uniform product particle size. This finding strongly supports the proposed La^3+^ slow-release dissolution-reprecipitation mechanism, which achieves an effect analogous to homogeneous precipitation in controlling nucleation and crystal growth.

**Figure 13:**
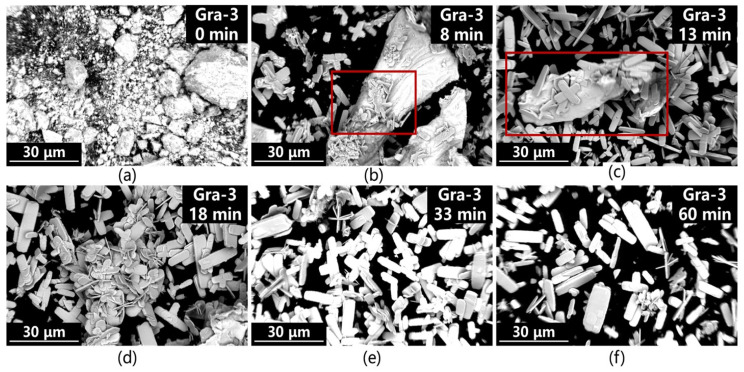
SEM analysis images of the gradient alkali conversion and carbonation process at different time points: (**a**) 0 min; (**b**) 8 min; (**c**) 13 min; (**d**) 18 min; (**e**) 33 min; (**f**) 60 min. The red square shows the heterogeneous nucleation of lanthanum carbonate on the precursor.

The morphological evolution of the products during the gradient alkaline carbonation process can be divided into three stages based on Figure 13a–f: (1) the initial stage (Figure 13a, 0 min, i.e., the alkali conversion precursor) features lanthanum hydroxide agglomerates; (2) in the intermediate stage (approx. 8 min), fusiform or cruciform crystal nuclei begin to appear on the precursor surface, gradually growing and detaching as the reaction proceeds; (3) in the final stage (60 min), the precursors are completely dissolved, leaving only well-defined fusiform flaky lanthanum carbonate crystals in the system. Throughout the process, the disappearance of lanthanum hydroxide crystals and the formation of lanthanum carbonate crystals can be observed, directly illustrating the effectiveness of the proposed gradient alkaline carbonation method in controlling the “dissolution-reprecipitation” process to achieve slow La^3+^ release.

**Figure 14:**
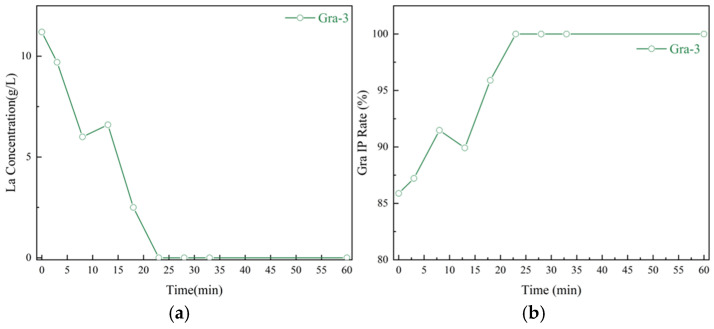
Time-dependent analysis of the gradient alkali conversion and carbonation process: (**a**) Real-time concentration of lanthanum ions; (**b**) Real-time precipitation rate.

The analysis results of La^3+^ content in the filtrate during the gradient alkali conversion carbonation process (Figure 14) further demonstrate the controllability of the La^3+^ release process. Specifically, the anomalous increase in the curve of La element content in the solution at 13 min precisely represents the dissolution phenomenon of La(OH)_3_ in the solution, partially elevating the La^3+^ content in the system. This anomalous point corresponds to the time when the particle size peak disappears, XRD indicates that lanthanum carbonate is dominant, and SEM reveals the dissolution of the bulk La(OH)_3_ precursor, all of which indicate that at this moment, the rapid dissolution of La(OH)_3_ releases a large amount of La^3+^, leading to an anomalous increase in La^3+^ concentration in the solution. Compared with the simultaneous alkali conversion carbonation process, the gradient alkali conversion carbonation process can ensure that La^3+^ in the system is maintained at a low supersaturation, thereby achieving controllability of crystal nucleation and growth. Regarding the quantitative description of “low supersaturation”, the present study provides only indirect evidence based on the convergence of particle size distribution and the real-time concentration profile; quantitative verification using in situ supersaturation sensors or theoretical modeling is recommended for future work.

Gra-3 is selected as the representative because it lies in the mid-range of alkali dosage and best captures the typical evolution of the gradient process. The anomalous rise in La^3+^ concentration at 13 min (arrow) corresponds to the rapid dissolution of the La(OH)_3_ precursor, which is key to maintaining low supersaturation and achieving homogeneous precipitation. Trends for other alkali dosage groups are similar and omitted for brevity.

The calculation method for the real-time precipitation rate in gradient alkali-to-carbonate conversion is the same as that in Formulas (2) and (3).

**Figure 15:**
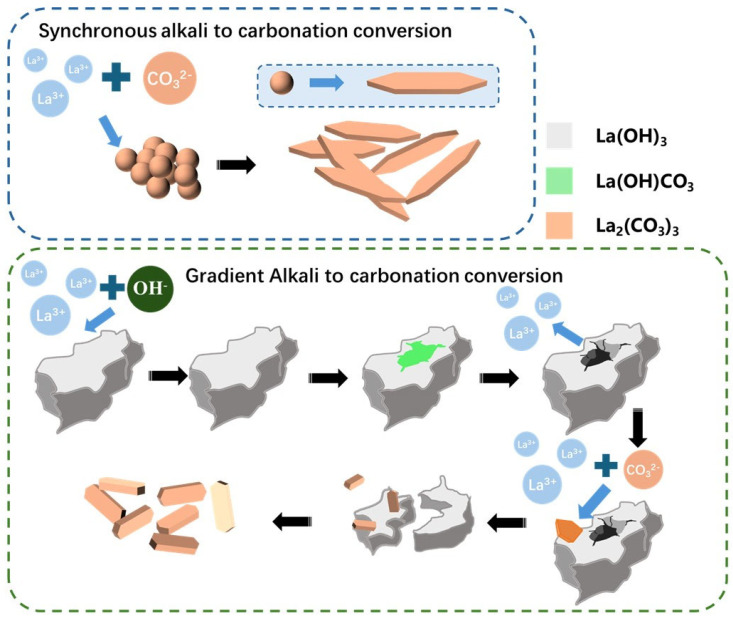
Comparison of the mechanisms of synchronous alkali conversion and carbonation and gradient alkali conversion and carbonation.

Based on the comprehensive analysis of the phase, morphological characteristics, and particle size distribution of the process products, the gradient alkaline conversion carbonation product is superior to the synchronous alkaline conversion carbonation product in terms of phase purity and particle size distribution. The fundamental reason lies in the gradient alkaline conversion carbonation method proposed in this paper, which controls the “precipitation-dissolution-reprecipitation” of La^3+^, as shown in Figure 15. Before carbonation, a lanthanum hydroxide precursor is preferentially formed, storing most of the La elements in the precipitate in the form of La(OH)_3_. In the subsequent carbonation process, by controlling the addition amounts of sodium hydroxide and CO_2_, the slow release of La^3+^ is controlled, maintaining the system at a low supersaturation level. This effectively inhibits rapid nucleation in a short time caused by instantaneous supersaturation, achieving an effect similar to homogeneous precipitation. Especially in rare earth chloride solutions with conventional industrial concentrations, the goals of large median particle size, narrow particle size distribution, and high phase purity of the product are achieved.

## 4. Conclusions

To address the challenges in traditional carbonation of rare earth chlorides—namely, the low feedstock concentration and the need for continuous adjustment of precipitant dosage to maintain stable pH, which complicate industrial-scale implementation—this study proposes a gradient alkaline carbonation method. The significant advantages and underlying mechanisms of this method in the preparation of lanthanum carbonate are systematically demonstrated. The results are as follows:

(1) The gradient alkaline carbonation method successfully yielded lanthanum carbonate tetrahydrate with a single-phase and well-developed crystalline structure, as evidenced by its sharp and clear XRD diffraction peaks. Furthermore, the product obtained via this method exhibited a relatively large median particle size of approximately 10 μm, a narrow particle size distribution, and Span = ((D90 − D10)/2D50) maintained at around 1.0.
(2) By rationally regulating the reaction pathway and the pH of the system, the gradient alkaline carbonation process preferentially forms a lanthanum hydroxide precursor. Subsequently, with continuous CO_2_ introduction and slow supplementary addition of alkali, a “precipitation-dissolution-reprecipitation” crystallization mechanism is facilitated. This allows for the slow and sustained release of La^3+^, effectively reducing the supersaturation of the reaction system and achieving an effect akin to homogeneous precipitation. Thermodynamic simulation using Visual MINTEQ provided a rational basis for selecting the pH control interval, thereby enabling the gradient design.

In summary, this study not only elucidates a universal mechanism for optimizing crystal growth through controlled ion release but also confirms, from an engineering perspective, that the gradient alkaline carbonation process can achieve nearly complete precipitation of rare earth ions from feedstock solutions at conventional industrial concentrations, with negligible residual rare earth ions in the filtrate. These findings provide a direct, practically valuable theoretical basis and technical pathway for the large-scale application of the novel green process coupling “rare earth chloride carbonation with sodium chloride electrolysis.”

It should be noted that the present study focuses on precipitation behavior and product quality. The suitability of the NaCl-containing filtrate for membrane electrolysis and its long-term recycling performance are not experimentally evaluated here; these aspects require dedicated investigation in future work.

## Figures and Tables

**Table 1:** Comparison of key results for different carbonation methods.

Method	Product Phase	D50 (μm)	Precipitation Rate (%)	Key Issue/Advantage
MgO	La_2_(CO_3_)_3_	10.75	88.83	Low precipitation rate
NaOH	La_2_(CO_3_)_3_	21.4	Not reported	Large size, but pH control difficult
Synchronous	Mixed hydrates	8.0–8.8	≥99.99	Complete precipitation, but impure phase and broad distribution
Gradient	Pure La_2_(CO_3_)_3_·4H_2_O	~10	≥99.99	Single phase, uniform size

**Table 2:** Reagents used in this study.

Reagent	Specification	Manufacturer	Purpose
Hydrochloric acid	Analytical reagent	Sinopharm	Preparation of LaCl_3_ solution
Sodium hydroxide	Analytical reagent	Sinopharm	Alkali conversion
Lanthanum chloride	–	Gansu Rare Earth	Raw material for carbonation
Lanthanum oxide	4N (99.99%)	Ganzhou Jinxigu	Preparation of LaCl_3_ solution
Carbon dioxide	≥99.50%	Beijing Shunan Qite	Carbonation

Research site: State Key Laboratory of Rare Earth Metallurgy and Materials, GRINM (Beijing General Research Institute for Nonferrous Metals), Beijing, China.

## Data Availability

The original contributions presented in this study are included in the article. Further inquiries can be directed to the corresponding authors.

## Abbreviations

Syn	Synchronous Alkali Conversion–Carbonation
Gra	Gradient alkali conversion–carbonation
